# Estimating the Impact of Tuberculosis Pathways on Transmission—What Is the Gap Left by Passive Case Finding?

**DOI:** 10.1093/infdis/jiae390

**Published:** 2024-08-06

**Authors:** Katherine C Horton, Ty McCaffrey, Alexandra S Richards, Alvaro Schwalb, Rein M G J Houben

**Affiliations:** TB Modelling Group, TB Centre, London School of Hygiene and Tropical Medicine, United Kingdom; Department of Infectious Disease Epidemiology, London School of Hygiene and Tropical Medicine, United Kingdom; TB Modelling Group, TB Centre, London School of Hygiene and Tropical Medicine, United Kingdom; Department of Infectious Disease Epidemiology, London School of Hygiene and Tropical Medicine, United Kingdom; TB Modelling Group, TB Centre, London School of Hygiene and Tropical Medicine, United Kingdom; Department of Infectious Disease Epidemiology, London School of Hygiene and Tropical Medicine, United Kingdom; TB Modelling Group, TB Centre, London School of Hygiene and Tropical Medicine, United Kingdom; Department of Infectious Disease Epidemiology, London School of Hygiene and Tropical Medicine, United Kingdom; Instituto de Medicina Tropical Alexander von Humboldt, Universidad Peruana Cayetano Heredia, Lima, Peru; TB Modelling Group, TB Centre, London School of Hygiene and Tropical Medicine, United Kingdom; Department of Infectious Disease Epidemiology, London School of Hygiene and Tropical Medicine, United Kingdom

**Keywords:** tuberculosis, subclinical, transmission, dynamics, detection

## Abstract

Current passive case-finding policies have not resulted in the expected decline in tuberculosis incidence. Recognition of the variety of disease pathways experienced by individuals with tuberculosis highlights how many are not served by the current prevention and care system and how much transmission is missed.

## Teaser

Recognition of the variety of disease pathways experienced by individuals with tuberculosis highlights how many are not served by the current passive case-finding system and how much transmission is missed, including transmission from subclinical disease.

Tuberculosis (TB) remains one of the largest infectious causes of mortality and morbidity globally [1]. TB prevention and care policies in past decades have relied heavily on passive case finding (PCF), where individuals with TB are expected to recognize symptoms and access care. Passive case-finding policies reflect an understanding that TB would progress linearly with mostly simultaneous onset of symptoms and infectiousness [2]. While millions of lives have been saved, transmission has persisted, with generalized transmission in the population driving incidence in high-burden settings [1].

In recent years, enduring assumptions around TB natural history have been shown to not hold. Many individuals have bacteriologically positive disease without awareness of symptoms, known as subclinical TB [3], and appear to contribute to transmission [4]. Analysis of historical and contemporary cohorts has also shown that TB is highly nonlinear, with individuals experiencing a wide range of pathways after infection [5, 6].

Here we combine insights on disease pathways with likely infectiousness to explore transmission across different pathways—based on disease trajectories, speed of progression, and eligibility for PCF services—and the contribution of subclinical TB within each of those pathways.

**Table 1.** Transmission Across Disease Pathways and Contributions to Transmission

Pathway	Proportion of Individuals Who Contribute to Transmission, % (UI)	Total Transmission		Transmission Within 24 Months of Infection	
		Proportion of Transmission, % (UI)	Proportion of Transmission Attributable to Subclinical Disease, % (UI)	Proportion of Transmission Within 24 Months of Infection, % (UI)	Proportion of Transmission Within 24 Months of Infection Attributable to Subclinical Disease, % (UI)
Trajectory					
Progression	20.4 (17.2–23.8)	23.0 (18.6–27.8)	25.9 (21.3–31.5)	48.9 (40.1–58.4)	32.0 (25.7–39.5)
Regression	35.5 (31.5–39.5)	14.9 (11.7–18.8)	77.5 (66.8–86.8)	52.5 (43.6–62.2)	82.6 (71.4–92.5)
Undulation	44.0 (39.7–48.4)	62.0 (56.4–67.1)	56.1 (50.9–61.7)	24.3 (20.4–28.5)	71.1 (62.8–79.1)
Time between infection and onset of infectious disease					
Rapid (≤24 mo)	68.5 (64.8–72.3)	71.1 (65.6–76.3)	52.2 (47.6–57.2)	48.1 (43.4–53.0)	60.8 (55.5–66.1)
Slow (>24 mo)	31.5 (27.7–35.2)	28.9 (23.7–34.4)	52.7 (45.9–60.2)	.0 (.0–.0)	.0 (.0–.0)
PCF eligibility					
Never PCF eligible	61.9 (57.7–65.8)	30.7 (26.1–35.5)	94.7 (93.4–96.0)	41.6 (36.1–46.9)	94.9 (92.6–96.8)
Pre-PCF eligible	38.1 (34.2–42.3)^a^	24.9 (22.3–27.5)	66.3 (63.4–68.9)	45.3 (39.7–51.5)	69.1 (64.5–73.1)
Post-PCF eligible		44.3 (39.5–48.9)	15.0 (11.6–18.8)	22.8 (17.4–28.0)	8.0 (4.1–12.5)
Total	100	100	52.4 (48.6–56.6)	34.2 (30.3–38.2)	60.8 (55.5–66.1)

Table shows proportion of individuals who contributed to transmission, categorized across disease pathways. Contributions to transmission are reported for total transmission and transmission within 24 months of infection, and the proportion of transmission attributable to subclinical disease is shown.

Abbreviations: PCF, passive case finding; UI, uncertainty interval.

^a^Pre- and post-PCF pathways are part of the same individual disease pathway, so are combined.

## METHODS

Our analyses builds on an existing compartmental cohort model [5], which follows individuals from *Mycobacterium tuberculosis* (*Mtb*) infection through noninfectious, subclinical (infectious but not recognizing symptoms), and clinical (infectious and recognizing symptoms) disease states. Transitions are informed by data from 25 historic cohorts of adults or adolescents with TB or recent exposure who were followed for at least 12 months without therapeutic intervention [7] and further constrained by data on disease state prevalence ratios, disease duration, and mortality [5]. We extended this model to reflect current PCF-based TB diagnosis and treatment for individuals with clinical disease, approximating 70% case detection of clinical disease [8–10]. See Supplementary Figure 1 for model structure and parameters.

We modeled 1000 cohorts of 10 000 individuals over 10 years from *Mtb* infection with 1-month time steps. For each cohort, we sampled transition parameters and relative infectiousness of subclinical TB (see Supplementary Table 1 for median and ranges). We did not explicitly model transmission within the model. Instead we assessed contributions to transmission by counting the number of months spent in the clinical disease state and the subclinical disease state over a period of 10 years following infection and assigning a relative infectiousness to subclinical TB compared with clinical TB. In the main analysis, we set the relative infectiousness for subclinical TB at a conservative estimate of 0.5 with a uniform uncertainty interval (UI) of 0.2 to 0.8, loosely informed by recent studies [4], and explored this in sensitivity analyses.

We defined 3 categories of mutually exclusive pathways to explore contributions to transmission by individuals who developed infectious disease within 10 years of *Mtb* infection. First, we examined trajectories over the 10-year course of disease: (1) *progression* included individuals who never reenter a previous disease state after reaching a more advanced state; (2) *regression* included individuals who reentered subclinical or noninfectious states after progressing to more advanced disease and never again progressed to a more advanced state; (3) *undulation* included individuals who progressed, regressed, and then progressed again between states (ie, those who experienced at least 2 changes of direction). Second, we classified individuals based on the time between infection and onset of infectious (subclinical or clinical) disease: *rapid* included individuals whose disease pathways included an infectious state within 24 months of infection, with the remainder classified as *slow*. Third, we based categories on whether individuals were eligible for PCF, defined in the main analysis as individuals who spent at least 2 consecutive months with clinical disease (in line with systematic reviews of self-reported duration of symptoms prediagnosis) [11, 12]. Within those who were PCF-eligible, we separated a pre- and post-PCF eligibility period informed by the PCF threshold (see Supplementary Figure 2 for an illustration of pathways). From these pathways, we further distinguished how much transmission occurred during time spent in subclinical disease and how much transmission occurred during the first 24 months after infection.

The contribution of pathways and periods was calculated as a proportion of total transmission. Median values and 95% UIs are reported.

To examine the sensitivity of our results to key model assumptions, we varied the impact of relative infectiousness for subclinical disease from 0 to 1.5 times that of clinical disease and lowered the threshold for PCF eligibility to either at the start or after the first month of clinical disease.

Data and analysis code can be found at GitHub (https://github.com/ERC-TBornotTB/Transmission).

## RESULTS

Overall, half (52.4% [95% UI, 48.6%–56.6%]) of transmission during the 10-year period came from individuals with subclinical disease (Table 1). In the primary analysis, individuals who experience undulating pathways contributed >60% of all transmission, the majority coming from subclinical disease. More than two-thirds (71.1% [95% UI, 65.6%–76.3%]) of transmission came from people who developed infectious disease within 24 months postinfection, who contributed relatively more to transmission as they spent more of the observation time in infectious states. Broadly, around a third of all transmission over the 10 years occurred within the first 24 months after infection (34.2% [95% UI, 30.3%–38.2%]), a similar proportion during the remaining 8 years among people who rapidly progressed (37.0% [95% UI, 32.0%–41.6%]), and just under a third from people who developed infectious disease >24 months after infection (28.9% [95% UI, 23.7%–34.4%]).

With these dynamics, we found that 61.9% (95% UI, 57.7%–65.6%) of individuals who experience infectious disease never become PCF-eligible. When combined with pre-PCF periods of those who do become PCF eligible, half of all transmission occurs before the health system is expected to be accessed, >80% of which is attributable to subclinical disease. Notably, of the transmission that occurs pre-PCF, just under half occurs within the first 24 months after infection (45.3% [95% UI, 39.7%–51.5%]).

Lowering the threshold for PCF had a limited impact on our findings (Supplementary Figure 3). If PCF eligibility started immediately after the onset of clinical disease, still around half of transmission had already occurred (44.6% [95% UI, 38.7%–50.9%]). These findings were robust to assumptions of relative infectiousness; even when assumed to be 0.25, just under half of transmission occurred during never-PCF-eligible and pre-PCF periods (Supplementary Table 2, Supplementary Figure 4). Conversely, if subclinical disease was assumed to be as infectious as clinical (ie, relative infectiousness was 1.0), only one-third of transmission occurred during PCF-eligible periods (33.5 [95% UI, 28.9%–38.0%]) (Supplementary Table 3, Supplementary Figure 4).

## DISCUSSION

By extending an existing model, we were able to carefully explore the role of varied disease pathways and transmission in the context of a well-functioning PCF-based TB program and to estimate the timing and pattern of transmission, including what is likely missed by current care systems. Our findings indicate that current PCF programs would miss at least half of transmission, even when meeting the threshold of a 70% case detection rate, and >80% of this missed transmission came from individuals experiencing subclinical transmission. In addition, although a third of transmission occurs within 24 months of infection, transmission happens over a prolonged period of disease, with many individuals moving in and out of infectious disease states throughout years before dying or recovering from TB, or receiving diagnosis and treatment in the PCF system.

Combined, these findings explain why the current PCF-based care system has not led to the expected decline in TB burden, as even when case detection targets are reached, transmission persists. In addition, the model fits with empirical observations, where randomized trials for population-wide screening found a major impact on burden and transmission when screening was “symptom-agnostic”—that is, individuals were screened regardless of reported symptoms [13]—whereas trials where screening relied on reported symptoms found limited to no impact [14].

Our analysis is model-based by necessity, as both recorded *Mtb* infection and onset of infectious disease would (rightly) lead to treatment. As with all models, our parameterization relies on assumptions, but values for underlying progression and regression were data-driven [5, 9], and newly introduced parameters were conservative and explored in sensitivity analyses. Our assumed relative infectiousness of 0.5 for subclinical TB was below the lower bound of recent estimates based on empirical data [4], and within the range of more distal indicators such as the relative proportion of individuals with positive sputum smear microscopy results [4]. While results did vary with relative infectiousness, even at a highly conservative value of 0.25, close to half of transmission was missed before PCF eligibility. We also assumed a well-functioning PCF system, where 70% of individuals with incident TB are diagnosed and receive effective treatment each year. Unfortunately, PCF programs perform less well in many high-burden settings where resources remain constrained and the amount of ongoing transmission is likely to be higher.

Transmission from subclinical disease appears to be a key driver for PCF systems not interrupting transmission. Efforts to interrupt transmission through treatment of disease will likely require repeated cycles of symptom-agnostic screening to fully break the persisting cycle of new infections and infectious disease pathways [13, 15]. Other strategies that prevent disease or transmission, including vaccination and TB preventive treatment, should also be considered.

## Supplementary Data

Supplementary materials are available at *The Journal of Infectious Diseases* online (http://jid.oxfordjournals.org/). Supplementary materials consist of data provided by the author that are published to benefit the reader. The posted materials are not copyedited. The contents of all supplementary data are the sole responsibility of the authors. Questions or messages regarding errors should be addressed to the author.

## Supplementary Material

jiae390_Supplementary_Data

## Notes

***Acknowledgments.*** Conceptualization: K. C. H. and R. M. G. J. H. Data collection: K. C. H. and T. M. Data curation: T. M. Formal analysis: K. C. H. and T. M. Writing—original draft: K. C. H. and T. M. Writing—review and editing: All authors. Supervision: R. M. G. J. H.

***Financial support.*** This work was supported by the European Research Council (grant number 757699 to K. C. H., T. M., A. S. R., A. S., and R. M. G. J. H.). K. C. H., A. S., and R. M. G. J. H. are also supported by the National Institutes of Health (grant number R-202309-71190), and K. C. H. and A. S. R. by the UK Foreign, Commonwealth and Development Office (“Leaving no-one behind: transforming gendered pathways to health for TB”).
