# Supplementary material for: Estimating the Impact of Tuberculosis Pathways on Transmission—What Is the Gap Left by Passive Case Finding?
Source: J Infect Dis. 2024 Aug 6;230(5):e1158–61. doi: 10.1093/infdis/jiae390 (PMC11566222; doi:10.1093/infdis/jiae390)
Supplement: jiae390_Supplementary_Data [file jiae390_supplementary_data.docx]

**SUPPLEMENTARY MATERIALS**

**Estimating the impact of tuberculosis pathways on transmission - what is the gap left by passive case-finding?**

Katherine C. Horton^1,2^, Ty McCaffrey^1,2^, Alexandra S. Richards^1,2^, Alvaro Schwalb^1,2,3^, Rein M.G.J. Houben^1,2^

**Correspondence:** Katherine C. Horton, Department of Infectious Disease Epidemiology, London School of Hygiene and Tropical Medicine, Keppel St, London, WC1E 7HT, United Kingdom ([katherine.horton@lshtm.ac.uk](mailto:katherine.horton@lshtm.ac.uk))

**
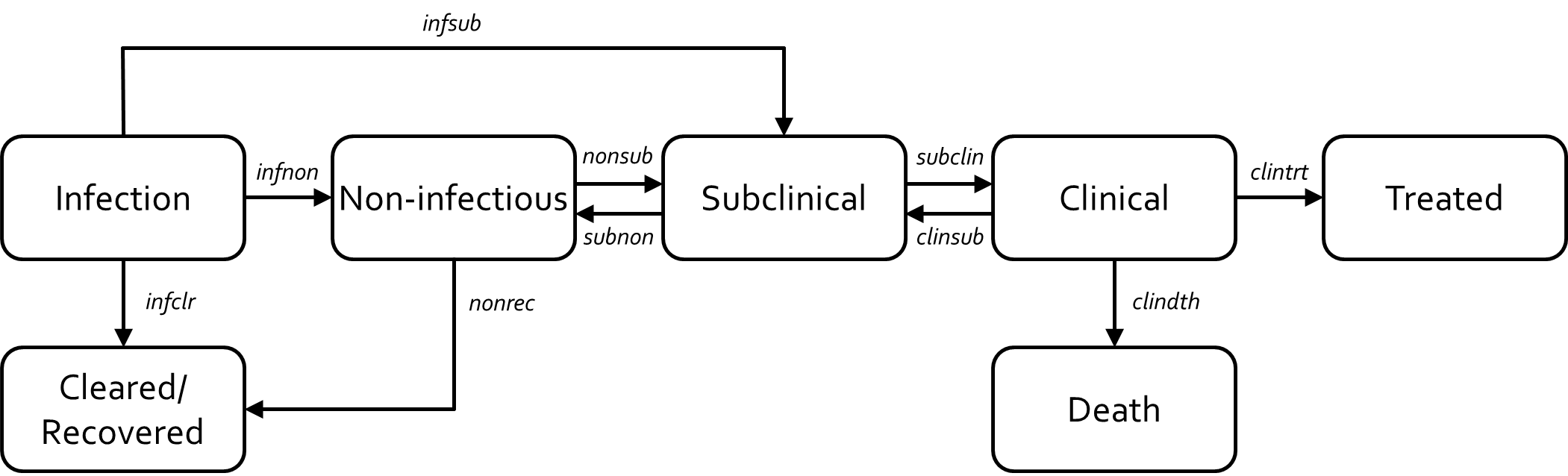
**

**Figure S1. Model structure.**

**Table S1. Model parameter ranges.** *Parameter values obtained from Horton et al. “Reevaluating Progression and Pathways Following Mycobacterium Tuberculosis Infection within the Spectrum of Tuberculosis” Proc Natl Acad Sci U S A 2023.*

| **Parameter** | **Description** | **Posterior median**  **(95%UI)** |
| --- | --- | --- |
| infclr | Infection to Cleared/Recovered | 1.83 (0.93 - 3.30) |
| infnon | Infection to Non-infectious | 0.10 (0.04 - 0.23) |
| infsub | Infection to Subclinical | 0.04 (0.01 - 0.10) |
| nonrec | Non-infectious to Cleared/Recovered | 0.18 (0.14 - 0.23) |
| nonsub | Non-infectious to Subclinical | 0.24 (0.21 - 0.28) |
| subnon | Subclinical to Non-infectious | 1.58 (1.24 - 2.03) |
| subclin | Subclinical to Clinical | 0.72 (0.56 - 0.94) |
| clinsub | Clinical to Subclinical | 0.57 (0.46 - 0.72) |
| clindth | Clinical to Death | 0.33 (0.28 - 0.38) |
| clintrt | Clinical to Treatment | 0.70 (0.60 - 0.80) |


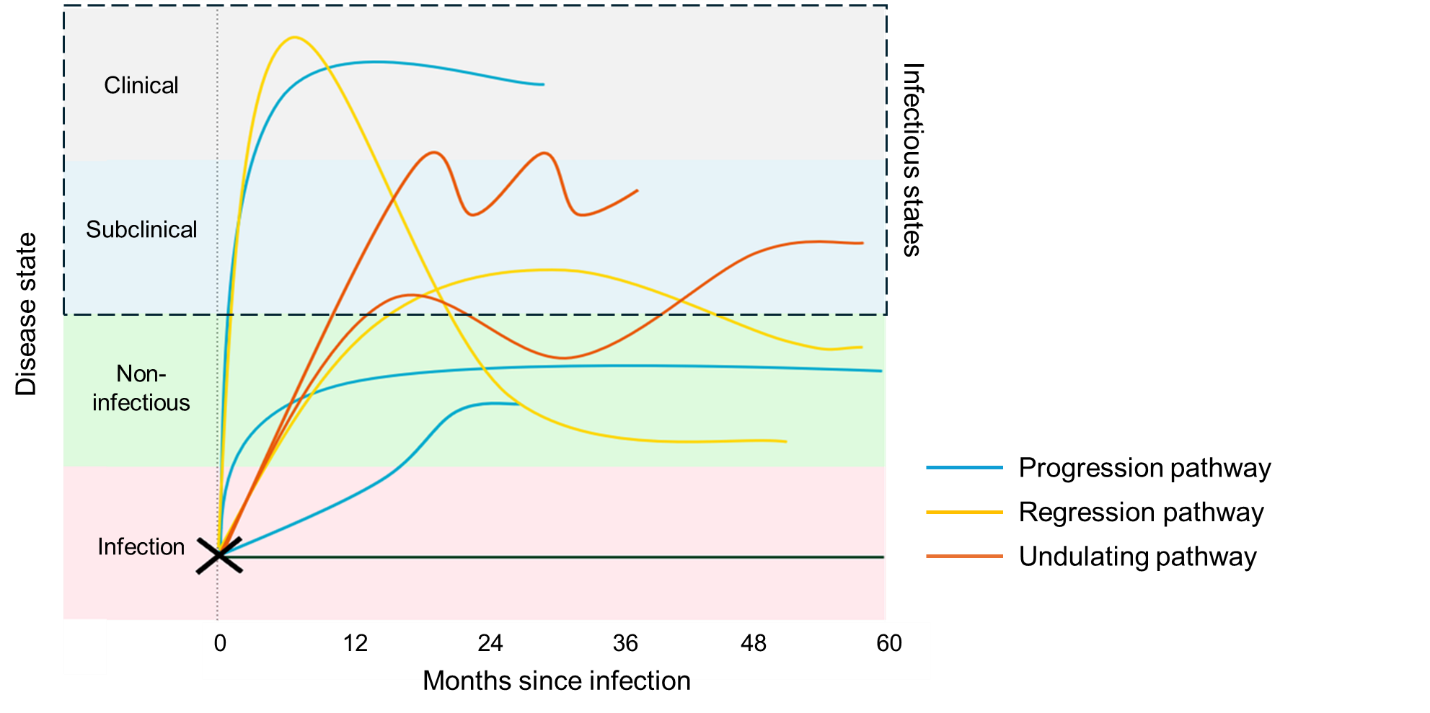


**Figure S2-A. Conceptual figure of natural history pathways according to changes in the direction of tuberculosis disease development.** *Figure shows categories based on trajectories over the 10-year course of disease: 1) PROGRESSION (represented in blue) included individuals who never re-enter a previous disease state after reaching a more advanced state; 2) REGRESSION (represented in yellow) included individuals who re-entered subclinical or non-infectious states after progressing to more advanced disease* *and never again progressed to a move advanced states; 3) UNDULATION (represented in red) included individuals who progressed, regressed, and then progressed again between states (i.e. those who experienced at least two changes of direction). Additionally, the figure highlights pathways categories based on the time between infection and onset of infectious (subclinical or clinical) disease: RAPID included individuals whose disease pathways included an infectious state within 24 months of infection, with the remainder classified as SLOW. (N.b., Changes in intensity within states is shown in this conceptual figure but were not modelled within the work described in this paper.)*


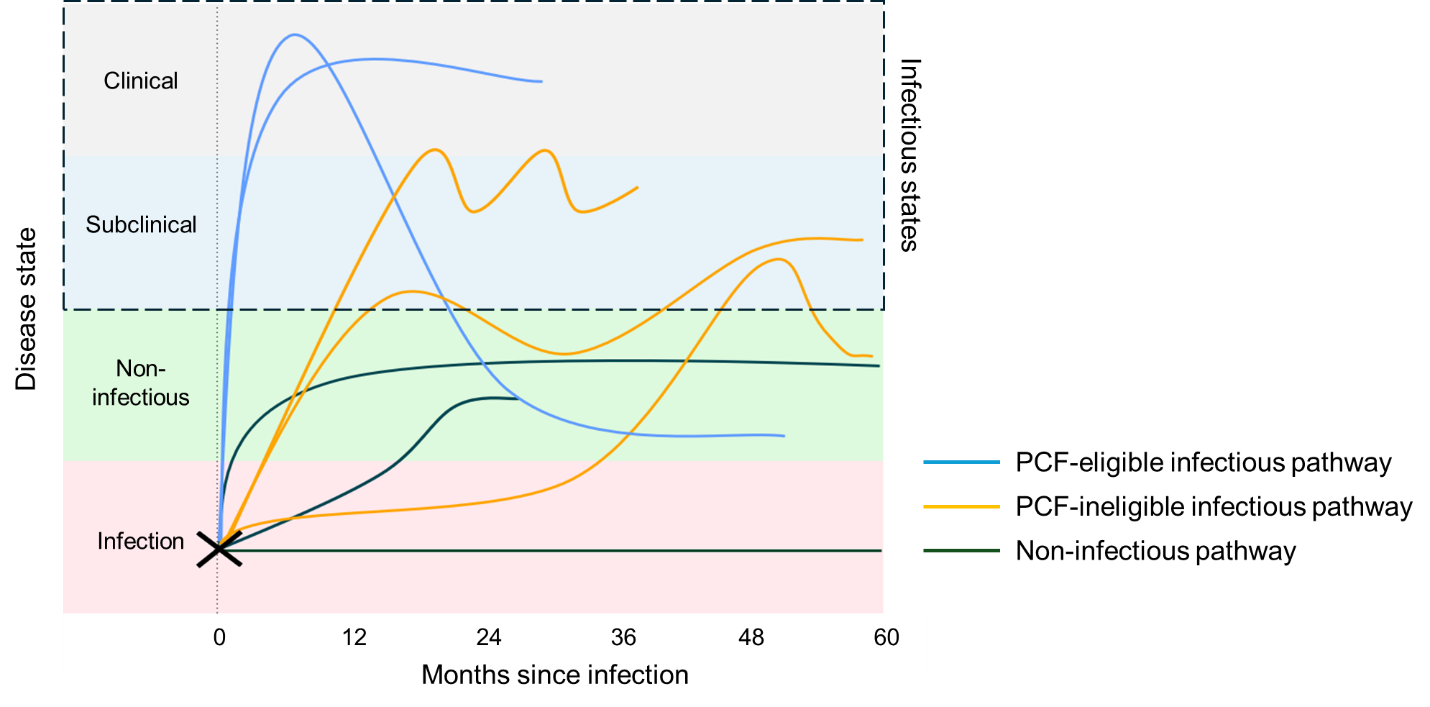


**Figure S2-B. Conceptual figure of natural history pathways according to passive case finding eligibility.** *Figure shows categories based on whether individuals were eligible for PCF, defined in the main analysis as individuals who spent at least two consecutive months with clinical disease. Within those who were PCF-eligible (represented in blue), we separated a PRE- and POST-PCF eligibility period informed by the PCF threshold. Additionally, the figure shows those who were PCF-ineligible (represented in yellow), i.e. individuals who never progressed to clinical disease, and those who traversed non-infectious pathways (represented in green), i.e. individuals who never progressed to infectious disease (subclinical or clinical). (N.b., Changes in intensity within states is shown in this conceptual figure but were not modelled within the work described in this paper.)*


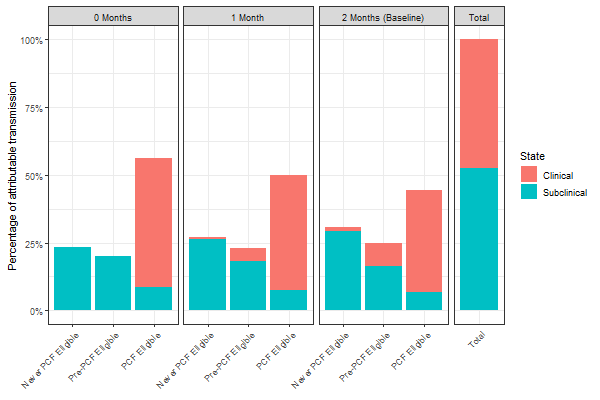


**Figure S3. Changing passive case-finding eligibility thresholds.** *Percentage of attributable transmission for passive case finding (PCF) pathways given by required consecutive months of clinical disease. PCF eligibility is defined as the number of consecutive months of clinical TB (0, 1, and 2 months) an individual is required to be eligible for detection by passive case-finding. Attributable transmission of individuals who never achieve PCF eligibility (never PCF eligible) within 10 years following infection is shown, as well as attributable transmission of those who do prior to becoming eligible (pre-PCF eligible) and following becoming PCF eligible (post-PCF eligible). Total transmission attributable to the cohort over the 10-year period is shown. Bars are shaded indicating which infectious TB state (clinical or subclinical) individuals occupied while contributing to transmission.*

**PCF threshold implementation:**

We implemented the PCF threshold independently of individuals being diagnosed, i.e. individuals are able to be diagnosed while in the clinical state, reflecting the real-life situation of spread in time to access care. The PCF threshold in the main body illustrates how a two month average delay affects transmission missed, and the sensitivity analyses show our results are quite robust to this threshold (see Figure S3).


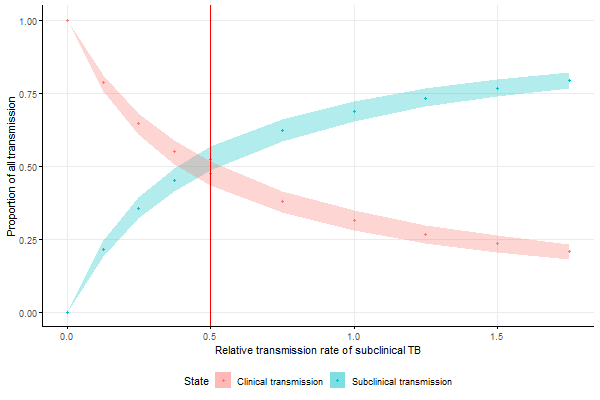


**Figure S4. Relative infectiousness of subclinical TB.**

**Table S2. Tuberculosis disease pathways and their contribution to transmission (relative infectiousness = 0.25, UI 0.1-0.4).**

| **Pathway** | **Proportion of individuals who contribute to transmission**  **(%, UI)** | **Total transmission** | | **Transmission within 24 months of infection** | |
| --- | --- | --- | --- | --- | --- |
|  |  | **Proportion of transmission  (%, UI)** | **Proportion of transmission attributable to subclinical disease**  **(%, UI)** | **Proportion of transmission within  24 months of infection (%, UI)** | **Proportion of transmission within  24 months of infection attributable to subclinical disease**  **(%, UI)** |
| **Trajectory** | | | | | |
| **Progression** | 20.4 (17.2 - 23.8) | 27.0 (21.9 - 32.7) | 14.9 (11.9 - 18.6) | 47.1 (38.2 - 57.2) | 19.2 (14.9 - 24.4) |
| **Regression** | 35.5 (31.5 - 39.5) | 12.3 (9.4 - 16.2) | 63.4 (51.0 - 76.9) | 50.4 (39.3 - 62.0) | 70.2 (54.7 - 86.1) |
| **Undulation** | 44.0 (39.7 - 48.4) | 60.4 (54.0 - 66.2) | 39.0 (34.1 - 44.6) | 21.7 (17.5 - 26.1) | 55.1 (45.7 - 65.4) |
| **Time between infection and onset of infectious disease** | | | | | |
| **Rapid (≤24 months)** | 68.5 (64.8 - 72.3) | 71.3 (65.1 - 76.9) | 35.3 (31.2 - 39.9) | 45.2 (40.1 - 50.7) | 43.6 (38.4 - 49.6) |
| **Slow (>24 months)** | 31.5 (27.7 - 35.2) | 28.7 (23.1 - 34.9) | 35.7 (29.7 - 43.0) | 0.0 (0.0 - 0.0) | 0.0 (0.0 - 0.0) |
| **PCF eligibility** | | | | | |
| **Never PCF eligible** | 61.9 (57.7 - 65.8) | 22.0 (18.3 - 26.1) | 89.9 (87.5 - 92.3) | 41.5 (36.0 - 47.1) | 90.2 (86.4 - 94.0) |
| **Pre-PCF eligible** | 38.1 (34.2 - 42.3)* | 22.6 (20.4 - 24.8) | 49.5 (46.4 - 52.9) | 44.4 (39.1 - 50.5) | 52.8 (47.5 - 57.9) |
| **Post-PCF eligible** |  | 55.5 (50.9 - 59.9) | 8.1 (6.1 - 10.4) | 23.5 (18.2 - 29.3) | 4.1 (2.0 - 6.6) |
| **Total** | 100 | 100 | 35.4 (31.9 - 39.3) | 32.3 (28.1 - 36.5) | 43.6 (38.4 - 49.6) |

*UI: Uncertainty interval; PCF: Passive case-finding.
* Pre- and post-PCF pathways are part of the same individual disease pathway, so are combined.*

**Table S3. Tuberculosis disease pathways and their contribution to transmission (relative infectiousness = 1.0, UI 0.4-1.6).**

| **Pathway** | **Proportion of individuals who contribute to transmission**  **(%, UI)** | **Total transmission** | | **Transmission within 24 months of infection** | |
| --- | --- | --- | --- | --- | --- |
|  |  | **Proportion of transmission  (%, UI)** | **Proportion of transmission attributable to subclinical disease**  **(%, UI)** | **Proportion of transmission within  24 months of infection (%, UI)** | **Proportion of transmission within  24 months of infection attributable to subclinical disease**  **(%, UI)** |
| **Trajectory** | | | | | |
| **Progression** | 20.4 (17.2 - 23.8) | 19.0 (15.4 - 23.2) | 41.2 (35.1 - 47.5) | 51.3 (43.0 - 60.5) | 48.6 (41.1 - 56.0) |
| **Regression** | 35.5 (31.5 - 39.5) | 17.3 (14.1 - 21.2) | 87.4 (80.6 - 92.9) | 53.9 (45.3 - 62.5) | 90.4 (83.3 - 96.0) |
| **Undulation** | 44.0 (39.7 - 48.4) | 63.6 (58.4 - 68.1) | 71.9 (67.4 - 76.2) | 26.6 (23.0 - 30.8) | 83.0 (77.1 - 88.3) |
| **Time between infection and onset of infectious disease** | | | | | |
| **Rapid (≤24 months)** | 68.5 (64.8 - 72.3) | 71.0 (66.2 - 76.1) | 68.6 (64.6 - 72.5) | 50.7 (46.5 - 55.5) | 75.6 (71.3 - 79.6) |
| **Slow (>24 months)** | 31.5 (27.7 - 35.2) | 29.0 (23.9 - 33.8) | 69.1 (62.6 - 75.2) | 0.0 (0.0 - 0.0) | 0.0 (0.0 - 0.0) |
| **PCF eligibility** | | | | | |
| **Never PCF eligible** | 61.9 (57.7 - 65.8) | 39.3 (34.2 - 44.6) | 97.2 (96.6 - 98.0) | 41.7 (36.2 - 47.1) | 97.4 (96.2 - 98.4) |
| **Pre-PCF eligible** | 38.1 (34.2 - 42.3)* | 27.1 (24.1 - 30.2) | 79.7 (77.5 - 81.6) | 46.1 (40.0 - 52.7) | 81.7 (78.3 - 84.6) |
| **Post-PCF eligible** |  | 33.5 (28.9 - 38.0) | 26.1 (20.8 - 31.6) | 21.3 (16.4 - 26.5) | 14.7 (7.8 - 22.3) |
| **Total** | 100 | 100 | 68.7 (65.2 - 72.1) | 36.0 (32.5 - 39.7) | 75.6 (71.3 - 79.6) |

*UI: Uncertainty interval; PCF: Passive case-finding.
* Pre- and post-PCF pathways are part of the same individual disease pathway, so are combined.*
